# Mucous membrane pemphigoid in a patient with hypertension treated with atenolol: a case report

**DOI:** 10.1186/1752-1947-6-373

**Published:** 2012-10-31

**Authors:** Patnarin Kanjanabuch, Samornroj Arporniem, Suparat Thamrat, Pannipa Thumasombut

**Affiliations:** 1 Department of Oral Medicine, Faculty of Dentistry, Chulalongkorn University, Bangkok, Thailand

## Abstract

**Introduction:**

Atenolol is commonly used by patients with hypertension, angina pectoris, or myocardial infarction. There have been reports of various adverse effects associated with the use of atenolol including bullous pemphigoid. To the best of our knowledge we present the first case report of atenolol-induced mucous membrane pemphigoid.

**Case presentation:**

A 42-year-old Thai man presented to our faculty after developing generalized fiery red gingiva and ulcerations on the buccal and labial mucosa after beginning atenolol treatment. Drug-induced mucous membrane pemphigoid was diagnosed from his clinical presentation and histopathologic and direct immunofluorescence examinations, combined with a history of beginning, and withdrawal, from atenolol therapy, with the lesions resolving after the cessation of atenolol therapy.

**Conclusions:**

To the best of our knowledge this is the first case of atenolol-induced oral mucous membrane pemphigoid reported in the literature. The observed lesions responded to withdrawal of the offending drug with complete remission. While drug-induced mucous membrane pemphigoid is an uncommon condition, dentists or other health care workers should include this condition in the differential diagnosis when a patient uses drugs suspected to be involved with drug-induced pemphigoid.

## Introduction

Atenolol is a synthetic β-1 selective adrenoreceptor-blocking agent. Current indications for its use include hypertension, angina pectoris, and myocardial infarction. The use of atenolol can induce various kinds of adverse mucosal effects including bullous or blistering drug eruptions, which are the most serious type of adverse drug reactions seen with this condition. Bullous drug eruptions may be classified as fixed drug eruptions, erythema multiforme, drug-induced pemphigus, or drug-induced pemphigoid. Mucous membrane pemphigoid (MMP) is a common immune-mediated subepithelial blistering disease mainly affecting the mucosa [1], often in the mouth. MMP is caused by the binding of autoantibodies to specific antigens such as bullous pemphigoid antigen 2 (BPAg2), and less often bullous pemphigoid antigen 1 (BPAg1) [2], laminin 5, laminin 6, α6-integrin subunit, β4-integrin subunit and collagen VII in the basement membrane zone [3]. This binding activates both leukocytes and complement, causing localized damage to the basement membrane, resulting in vesicle formation under the epithelium [4]. The initiating factor for the autoimmune response in MMP is usually unknown, but MMP is occasionally induced by the ingestion or local use of certain drugs. Drug-induced mucous membrane pemphigoid presents clinical, histologic, and immunopathologic features identical or closely similar to those of idiopathic pemphigoid disease, but patients with drug-induced pemphigoid are commonly younger than patients with idiopathic pemphigoid [5]. The systemic drugs implicated in causing drug-induced MMP include thiol compounds such as d-penicillamine and anti-hypertensive drugs such as clonidine, practolol and nadolol.

## Case presentation

A 42-year-old Thai man was referred to the Oral Medicine Clinic, Faculty of Dentistry, Chulalongkorn University, Thailand for the evaluation of ulcers in the mouth. The lesions had been present for more than six months and had been diagnosed as gingivitis.

**Figure 1:**
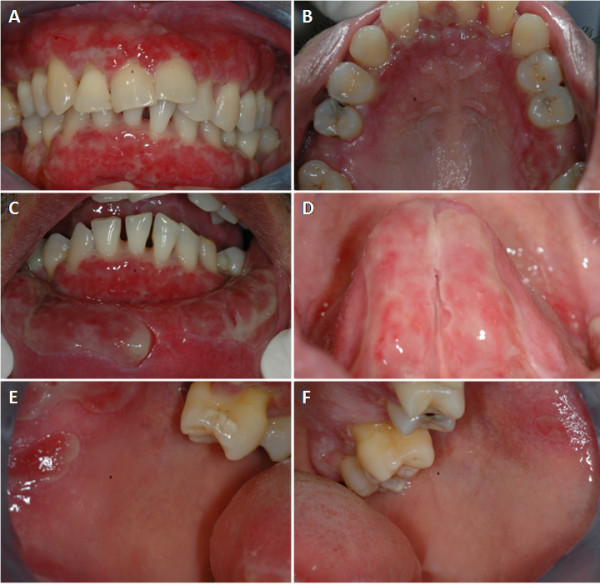
**Clinical findings at our patient’s first visit.** The gingival mucosa showed generalized erythema, desquamation, and multiple large ulcers covered with yellowish slough tissue (**A,B**). Multiple disrupted bullae were present on the labial mucosa (**C**), ventral surface of the tongue (**D**) and buccal mucosa (**E,F**).

His medical history was significant, with a diagnosis of hypertension and diabetes mellitus for a year. He had been treated with atenolol at 50mg per day, for one year and enalapril at 10mg per day for one week. He was on a controlled diet for diabetes mellitus. He did not smoke or drink and his medical history was otherwise unremarkable. Our patient’s extra-oral examination (skin, genitalia, eyes) was unremarkable. An intra-oral examination revealed generalized erythema, desquamation, and multiple large ulcers of the gingival mucosa covered with yellowish slough tissue. We found multiple disrupted bullae on the labial mucosa, buccal mucosa, and ventral surface of the tongue (Figure 1). Our patient was experiencing severe pain from these lesions and could not clean his mouth well or perform proper oral hygiene.

Our patient’s blood test results revealed normal range findings for complete blood count, erythrocyte sedimentation rate, creatinine and liver function tests (aspartate aminotransferase and alanine aminotransferase). Based on our patient’s history and examination findings, the differential diagnosis included allergy induced by chlorhexidine mouthwash, erythema multiforme, or vesiculobullous lesions.

Our patient was misusing chlorhexidine mouthwash by rinsing his mouth with it for longer than 10 minutes, so at our patient’s first visit, he was asked to stop the use of chlorhexidine mouthwash and was given diphenhydramine elixir mouthwash as a palliative treatment. This change resulted in the relief of pain after one week; however, the oral lesions were unchanged.

An incisional biopsy was taken from the tissue adjacent to the ulceration on the left buccal mucosa for histopathologic and direct immunofluorescence studies. The histopathological section of the biopsy was stained with hematoxylin and eosin (H&E). H&E staining showed an ulcerated parakeratinized stratified squamous epithelium overlying fibrovascular connective tissue. The underlying connective tissue was edematous, and was infiltrated by acute and chronic inflammatory cells, including neutrophils, eosinophils, plasma cells, and lymphocytes. These features indicated a non-specific subacute inflammation.

**Figure 2:**
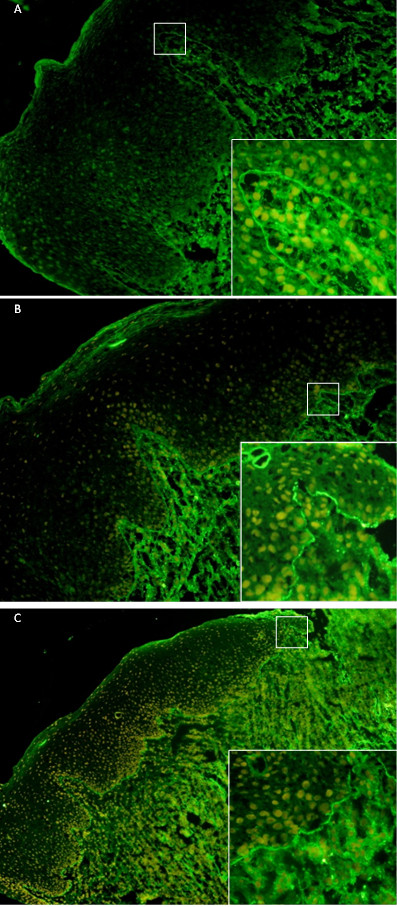
**Direct immunofluorescence showing deposition of IgG (A), IgA (B) and C3 (C) in a homogeneous linear pattern at the epithelial-connective tissue junction.** The boxed area is shown in the inset.

Direct immunofluorescence showed the deposition of IgG, IgA, and C3 in a homogeneous linear pattern at the dermoepidermal junction (Figure 2A-C). These findings can be associated with bullous pemphigoid, mucous membrane pemphigoid, bullous systemic lupus erythematosus, and epidermolysis bullosa acquisita. Our patient was given prednisolone 20mg every other day, with the dose increasing to a final level of 50mg per day after six weeks, with the lesions slightly improved; we then decided to stop prednisolone at the eighth week.

From the overall history, clinical presentation, result of direct immunofluorescence and slight response to prednisolone, we suspected a drug-induced condition, which might be caused by our patient’s current medication. Our patient was referred to his physician to discuss changing medications or to stop atenolol treatment altogether. Our patient discontinued atenolol use and was given enalapril at 10mg per day instead. We recommended the use of sodium bicarbonate mouthwash to our patient.

**Figure 3:**
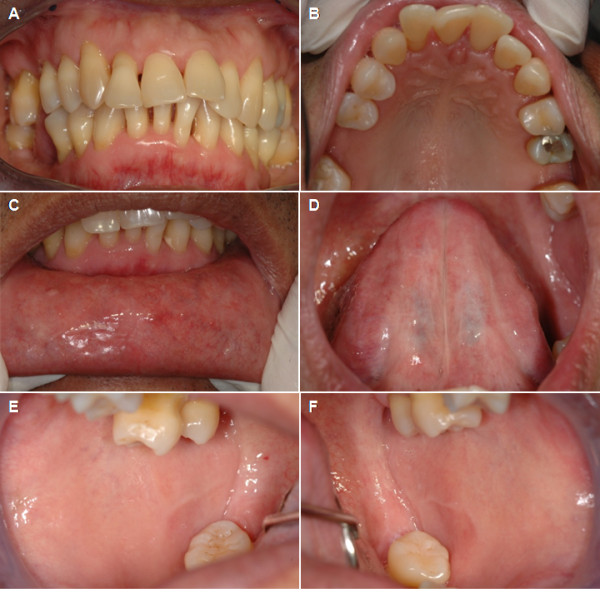
**Clinical findings at two-year follow-up.** All lesions were in complete remission. The gingiva (**A,B**), labial mucosa (**C**), ventral area of the tongue (**D**) and buccal mucosa (**E,F**) all have a normal appearance.

At one-month follow-up after atenolol cessation, the oral lesions showed a dramatic improvement; however, asymptomatic desquamative lesions of the attached gingiva remained. Our patient continued using sodium bicarbonate mouthwash. At three-month follow-up, all lesions were clear. We continued to follow-up our patient at six months, and one and two years with no recurrence of lesions observed. Figure 3 shows the patient’s oral condition at the two-year follow-up.

## Discussion

Drug-induced pemphigoid is the term used to describe cases presenting with clinical, histologic, and immunopathologic findings similar to idiopathic (autoimmune) pemphigoid but where the lesions develop subsequent to taking a drug. Previous reports have suggested various kinds of drugs such as anti-hypertensive drugs (especially those containing a thiol group), diuretics (especially furosemide), penicillamine, penicillin-derived antibiotics, sulfasalazine, phenacetin, and topical medications can cause bullous pemphigoid [5]. Penicillamine involved drug-induced pemphigoid frequently results in oral mucosal or cutaneous lesions [6]. There have been pathogenic theories postulated for drug-induced pemphigoid, one of which is that the drugs act as haptens and bind to proteins in the lamina lucida zone changing their antigenic properties, inducing anti-basement membrane zone antibodies, resulting in an autoimmune response [7]. Another pathogenic theory is that the drugs interact with suppressor/cytotoxic cell (CD8), decreasing suppressor cell activity, resulting in the hyper-production of autoantibodies [5].

The clinical manifestations of drug-induced pemphigoid are similar to those seen with cicatricial pemphigoid or mucous membrane pemphigoid. Drug-induced pemphigoid is more common in younger patients than is the idiopathic type. In drug-induced pemphigoid the bullous lesions erupt involving the mucous membranes immediately after taking the offending drug. Spreading of the bullous eruptions can then occur, which can appear similar to erythema multiforme [5]. The histologic features of pemphigoid are characterized by a split between the surface epithelium and underlying connective tissue (subepithelial separation) with numerous inflammatory cells present in the lesional area. If a biopsy captures the bullous lesion in its entirety, a tense dome-shaped uni-locular subepithelial blistering containing fibrin, edema fluid, and numerous inflammatory cells can be seen. Eosinophils typically predominate in the lesion, although neutrophils, lymphocytes, and histiocytes are commonly present [8]. Drug-induced pemphigoid lesions are often rather atypical in appearance. There may be intra-epithelial vesiculation with keratinocyte necrosis, with lymphocytes mainly seen in the connective tissue infiltrate [9].

In our case, histologic sections from peri-lesional areas showed an ulcerated parakeratinized stratified squamous epithelium overlying fibrovascular connective tissue. The underlying connective tissue was edematous, and was infiltrated by acute and chronic inflammatory cells, including neutrophils, eosinophils, plasma cells, and lymphocytes. These specimens exhibited features of a non-specific subacute inflammation. However, these observations were not conclusive.

Immunofluorescence studies are important tools in the investigation of autoimmune bullous disorders and are standard procedure for making an accurate diagnosis. The pemphigoid family (bullous pemphigoid, cicatricial pemphigoid, and herpes gestationis), epidermolysis bullosa acquisita, and bullous systemic lupus erythematosus are diseases characterized by the linear deposition of autoantibodies recognizing various target antigens along the basement membrane [10]. Bullous pemphigoid and cicatricial pemphigoid typically localize both IgG and C3 at the basement membrane (IgA and IgM may be seen) but epidermolysis bullosa acquisita and bullous systemic lupus erythematosus usually have multiple classes of immunoreactants. Epidermolysis bullosa acquisita exhibits extensive IgG/C3 staining with less IgA and IgM seen at the basement membrane. Bullous systemic lupus erythematosus is characterized by linear or granular IgG/IgA patterns, and C3, and can be positive or negative for IgM along the basement membrane [11].

The result of a direct immunofluorescence study of the biopsy sections of our patient showed deposition of IgG, IgA, and C3 in a homogeneous linear pattern at the dermoepidermal junction, which did not allow for discrimination between the conditions previously mentioned. To clearly distinguish among these groups, we considered the direct immunofluorescence results in conjunction with the clinical findings and patient history. We initially ruled out epidermolysis bullosa, although oral lesions are most commonly observed in dystrophic forms. However, oral lesions are uncommon in the absence of cutaneous lesions. In addition, our patient had no family history or initial lesions (vesicle or bullae) in areas easily exposed to low-grade trauma in early life. In our patient’s case, he presented with the initial eruption of lesions, with no history of repeated cycles of scarring resulting in microstomia, ankyloglossia or stricture esophagus. Bullous systemic lupus erythematosus was eliminated as our patient had no skin lesions or other organ involvement. We next considered the pemphigoid family, such as mucous membrane pemphigoid or cicatricial pemphigoid, because of the presence of oral mucosal lesions (generalized erythema, desquamation, multiple large ulcers covered with yellowish slough tissue along the gingiva, and multiple tense bullae of the oral mucosa and tongue). While bullous pemphigoid typically presents with skin lesions such as taut blisters, pruritus, papulovesicular or urticarial plaques on the flexor side of the extremities, oral mucosal involvement is uncommon [12]. After we observed the slight response of our patient to systemic steroids we then stopped prednisolone. We suspected his condition may be due to his medication. We then decided to refer our patient to his physician to consult about changing or discontinuing atenolol therapy, which he had taken for approximately six months prior to the eruption of lesions. A definitive diagnosis of ‘atenolol-induced mucous membrane pemphigoid’ was made due to the spontaneous remission of the lesions after our patient stopped taking atenolol.

There have been few cases of atenolol-induced bullous pemphigoid reported. In 1987, one report showed atenolol-induced blisters on the legs and trunk of a 59-year-old man [5] and in 2009, a retrospective medical history study of patients from northern Greece who had bullous pemphigoid, indicated one in 34 patients had received atenolol [13]. However, to the best of our knowledge, our case is the first report of atenolol-induced oral mucous membrane pemphigoid in the literature.

## Conclusions

Atenolol is a commonly used drug that can cause various adverse effects. To the best of our knowledge, no previous report has described atenolol-induced oral mucous membrane pemphigoid. A diagnosis of atenolol-induced mucous membrane pemphigoid was made from the history, clinical presentation, histopathologic and immunofluorescence results, and the response following drug therapy cessation. Examination of our patient and diagnostic assays initially suggested a diagnosis of idiopathic pemphigoid. However, when he did not response to systemic steroid therapy, a diagnosis of ‘drug-induced oral mucous membrane pemphigoid’ was made. Dramatic improvement after cessation or changing the medication is diagnostic for these lesions. Thiol and penicillamine drugs, and also atenolol, can cause mucous membrane pemphigoid. Physicians and other health care workers should recognize this side effect for correct early detection and proper management.

## Consent

Written informed consent was obtained from the patient for publication of this case report and accompanying images. A copy of the written consent is available for review by the Editor-in-Chief of this journal.

## Competing interests

The authors declare that they have no competing interests.

## Authors’ contributions

PK performed the clinical evaluations, diagnosis, treatments and was a major contributor in writing the manuscript. SA, ST and PT assisted in the treatments and wrote the manuscript. All authors read and approved the final manuscript.
